# A Comparative Study of Additive and Subtractive Manufacturing Techniques for a Zirconia Dental Product: An Analysis of the Manufacturing Accuracy and the Bond Strength of Porcelain to Zirconia

**DOI:** 10.3390/ma15155398

**Published:** 2022-08-05

**Authors:** Joon-Mo Moon, Chang-Sub Jeong, Hee-Jeong Lee, Ji-Myung Bae, Eun-Joo Choi, Sung-Tae Kim, Young-Bum Park, Seung-Han Oh

**Affiliations:** 1 Department of Dental Biomaterials and the Institute for Biomaterials and Implant, College of Dentistry, Wonkwang University, Iksan 54538, Korea; 2 Department of Dental Laboratory Technology, Faculty of Health and Medical Sciences, Wonkwang Health Science University, Iksan 54538, Korea; 3 Department of Oral & Maxillofacial Surgery, College of Dentistry, Wonkwang University, Iksan 54538, Korea; 4 Department of Periodontology, Dental Research Institute, School of Dentistry, Seoul National University, Seoul 03080, Korea; 5 BK21 Plus Project, Oral Science Research Center, Department of Prosthodontics, Yonsei University College of Dentistry, Seoul 03722, Korea

**Keywords:** additive manufacturing (AM), subtractive manufacturing (SM), zirconia, manufacturing accuracy, bond strength

## Abstract

This study was aimed at preparing zirconia samples via additive manufacturing (AM) and subtractive manufacturing (SM) and testing the following aspects: (1) the manufacturing accuracy of the zirconia samples and (2) the bond strength of porcelain to zirconia to evaluate the applicability of the zirconia fabricated by AM in dental clinics. We used three milling machines for SM (AR, K5, and UP) and a 3D printer for AM (AO). The manufacturing accuracy of the zirconia specimen in the internal and marginal areas was evaluated by superimposing techniques to calculate the root mean square (RMS) values. The bond strengths of porcelain to zirconia prepared via SM and AM were measured using a universal testing machine. The internal and marginal RMS values of the zirconia prepared by AM (AO) were within the range of those of the zirconia prepared by SM (AR, K5, and UP). Moreover, the bond strength value of the zirconia prepared by AM (35.12 ± 4.09 MPa) was significantly higher than that of the zirconia prepared by SM (30.26 ± 5.20 MPa). Therefore, AM technology has significant potential for applications in dentistry.

## 1. Introduction

Digital technology has recently been incorporated into analog-based dental technology. New methods for manufacturing digital dental products have been developed since the introduction of computer-aided design (CAD)/computer-aided manufacturing (CAM) systems in dentistry [1]. CAD/CAM-systems-based dental technologies are characterized by simplified manufacturing processes, which ensure a minimal deformation of the dental prosthesis that frequently occurs in the conventional complex multistep process [2].

According to the manufacturing methods, CAD/CAM systems are classified as subtractive manufacturing (SM) and additive manufacturing (AM) techniques. SM is widely used to prepare digital dental products, such as zirconia prostheses, with complicated conventional manufacturing methods [3,4]. SM technology demonstrates the advantages of a continuous fabrication process; however, the manufacturing accuracy in SM technology is affected by the wear of milling burrs, local fracture of the prosthesis, and the limitation of the tool path of the burrs [5,6].

Numerous AM technologies have been proposed recently in dentistry, such as stereolithography, fused deposition modeling, and selective laser sintering (SLS), since the introduction of 3D model production technology by Kodama in 1981 [7,8,9,10]. Polymer-based AM technologies are primarily used to produce provisional dentures, artificial teeth, and implant surgical guides with biocompatible 3D printing materials [11,12]. Moreover, metal-based AM technology has recently been developed to produce crowns, partial denture frames, surgical instruments, and oral/maxillofacial implants. However, AM technology is limited by the high cost of its equipment and materials [13]. In addition, ceramic-based AM technologies such as SLS and selective laser melting (SLM) are not suitable for powder-based 3D printers because the nonconductive properties of ceramics result in localized thermal gradients under laser irradiation, thermal shock as a result of the rapid heating–cooling process, and low dispersibility [14,15,16]. Recently, a zirconia-based slurry for AM was developed to produce ceramic dental prostheses. Slurry-based 3D technology has demonstrated significant potential for overcoming the limitations of SM, such as the considerable reduction in machinable blocks and burr replacement costs.

Zirconia has been known as one of the representative ceramic biomaterials since it was first proposed for use in the medical field in 1969 [17]. Zirconia has the potential to replace the alumina and titanium that is used for dental and orthopedic prostheses. In particular, the mechanical strengths of zirconia for orthopedics prepared via AM and SM technologies were similar. Denry et al. reported that the value of the bend strength of AM-fabricated zirconia was within the range of values of SM-prepared zirconia (800~1000 MPa) [18]. Many studies have evaluated the mechanical properties of zirconia prostheses fabricated with AM technology [19,20,21]. In addition, owing to its natural color, zirconia offers an advantage over metal-based dental prostheses in terms of esthetic restoration. Two types of zirconia are used in dentistry: full zirconia and porcelain-fused zirconia (PFZ). High-strength full zirconia is mainly used for posterior dental prostheses that require mechanical strength, and PFZ is used for anterior dental prostheses to improve esthetics. Measuring the bonding strength between zirconia and porcelain is essential to evaluate the mechanical properties of PFZ. Therefore, to assess the applicability of zirconia manufactured by additive manufacturing as a dental prosthesis, it is necessary to estimate the bonding strength of porcelain to zirconia.

In addition, for zirconia manufactured with the AM technology to be used in dental practice, the zirconia-based dental prosthesis must have micro-level accuracy to ensure a perfect fit with the abutment and prevent secondary caries in the oral cavity. Several studies have evaluated the accuracy of zirconia dental prostheses manufactured with SM technology [22,23,24]. Most of these studies have focused on the internal and marginal fitting accuracy between the abutment and crown prepared by SM technology, and the fitting accuracy was better than those of the dental prostheses manufactured via conventional dental technology; however, few studies have focused on the manufacturing accuracy of zirconia dental prostheses based on AM technology. Therefore, a study on the accuracy of the dental product prepared via AM technology is necessary.

The objective of this study was to estimate the manufacturing accuracy of zirconia dental products prepared via the AM and SM technologies. In addition, we compared the bond strength of porcelain to zirconia produced via the AM and SM technologies. We also examined the feasibility of using AM technology for producing zirconia prostheses for use in dentistry.

## 2. Materials and Methods

### 2.1. Materials

We used three milling machines for SM and one digital light processing (DLP)-type 3D printer for AM to prepare the zirconia specimens. Furthermore, we used a zirconia blank for SM and a zirconia slurry for AM. Detailed information on the devices and materials used in this study is listed in Table 1.

### 2.2. Accuracy Evaluation of the Internal and Marginal Areas

**Figure 1:**
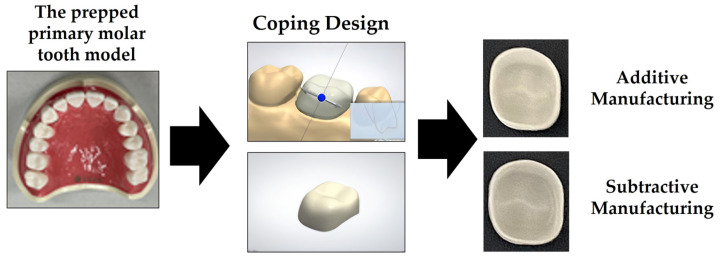
Schematic of the design and preparation of the zirconia specimens used in this study.

Figure 1 briefly illustrates the procedure for designing and preparing the zirconia specimens used in this study. First, we prepared the CAD reference data (CRD) by designing the specimens in the preparation area using dental design software (3 Shape Dental System, 3 Shape A/S, Copenhagen, Denmark) after scanning a prepped primary molar tooth model (D85DP-500B.1, Nissin Dental Product Inc., Kyoto, Japan). We then prepared the zirconia specimens based on the CRD using the three milling machines and one 3D printer. The prepared zirconia specimens were sintered in accordance with the instructions specified by the manufacturer. We then scanned the sintered zirconia specimens using a model scanner (Identica Blue, Medit Co., Seoul, Korea) to obtain the CAD test data (CTD).

**Figure 2:**
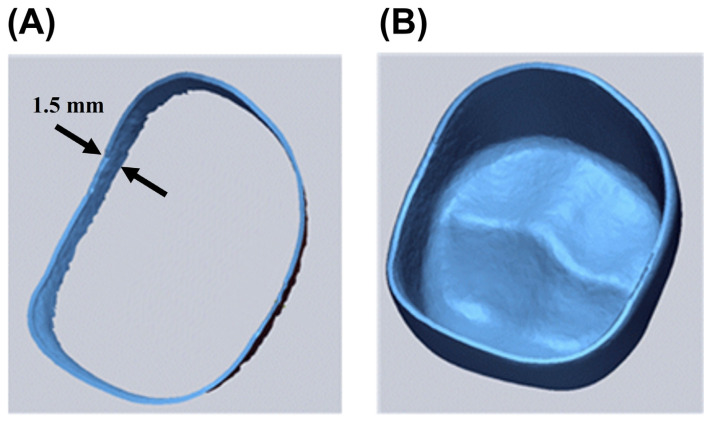
Images of (**A**) the marginal area and (**B**) the internal area obtained from the CAD reference data of the zirconia specimen.

To evaluate the accuracy of the zirconia specimens prepared by AM and SM, we superimposed the CTD and CRD using 3D image analysis software (Geomagic Control X, 3D Systems Inc., Rock Hill, SC, USA). We then calculated the distance between the points on the two image surfaces as root mean square (*RMS*) values [25,26]. The following formula, Formula (1), was used to calculate the *RMS* value:

(1) $$RMS=\frac{1}{\sqrt{n}}\times \sqrt{\sum_{i=1}^{n}{\left(X_{1,i}-X_{2,i}\right)}^{2}}$$

where *X*_1,*i*_ is the i-th point in the CRD, *X*_2,*i*_ is the i-th point in the CTD, and *n* denotes the total number of points measured for *RMS* analysis. The marginal area accuracy was evaluated by separately extracting 1.5 mm from the marginal end in the image of the zirconia specimen along the inner direction, as shown in Figure 2A. The internal area accuracy was estimated by the RMS values obtained from the entire area inside the zirconia specimen (Figure 2B). Furthermore, color-coded maps of the entire internal area were generated to visualize the magnitude and direction of the deviation between the CTD and CRD. Ten specimens from each group were used to evaluate the accuracy of the internal and marginal areas.

### 2.3. Test of the Bond Strength of Veneered Porcelain to Zirconia

To assess the bond strength of porcelain to zirconia that was prepared by AM and SM, we used the debonding/crack-initiation test specified in the International Standard, “ISO 9693-2 Dentistry―Compatibility testing―Part 2: Ceramic-ceramic systems” [27]. We used a milling machine (K5) for SM and a 3D printer (AO) for AM to prepare zirconia test specimens. First, large zirconia blocks (length, width, and thickness of 25.0 ± 1.0, 4.0 ± 0.1 and 10.0 ± 1.0 mm, respectively) were prepared by AM and SM methods. The zirconia blocks were then cut and trimmed to the scale required for a three-point flexural test (length, width, and thickness of 25.0 ± 1.0, 3.0 ± 0.1 and 0.50 ± 0.05 mm, respectively) using a high-precision cutting device (Accutom-100, Struers Inc., Cleveland, OH, USA). After the cutting process, the cut surface of the zirconia specimen was polished with silicon carbide paper (# 600) to minimize defects. Subsequently, we built a porcelain layer (with length, width, and thickness of 8.0 ± 0.1, 3.0 ± 0.1, 1.1 ± 0.1 mm, respectively) in the middle of the zirconia specimen using porcelain powder (IPS e.max Ceram/TI 2, Ivoclar Vivadent, Schaan, Liechtenstein). The porcelain layer was built by following the instruction manual provided by the manufacturer: after mixing the liquid provided by the manufacturer and porcelain powder to make a slurry, the porcelain slurry was layered in the center of the zirconia specimen by a hand instrument, and the moisture of the layered zirconia was removed using a paper tissue. The zirconia specimens with porcelain buildup were then heat treated at the sintering temperature recommended by the manufacturer. Ten specimens from each group were prepared for bond strength testing. The test of the bond strength of veneering porcelain to zirconia was performed using a universal testing machine (Instron 3345, Instron, Norwood, MA, USA) with a crosshead speed of 1.5 ± 0.5 mm/min. We also observed the debonded surface using a scanning electron microscope (SEM; Cube-II, Emcrafts Co., Ltd., Gwangju, Gyeonggi-do, Korea) and captured the elemental mapping of the debonded surface using an energy-dispersive X-ray spectroscope assembled in the SEM.

### 2.4. Statistical Analysis

The accuracy and bond strength test values were expressed as mean ± standard deviation. We analyzed the RMS values of the accuracy using one-way analysis of variance and the Games–Howell test as a post-hoc test (SPSS Ver. 23.0, IBM Co., Armonk, NY, USA). We also analyzed the bond strength results using an independent t-test. All statistical analyses were performed at a significance level of 95% (α = 0.05).

## 3. Results

### 3.1. Accuracy Evaluation of the Internal and Marginal Areas

**Figure 3:**
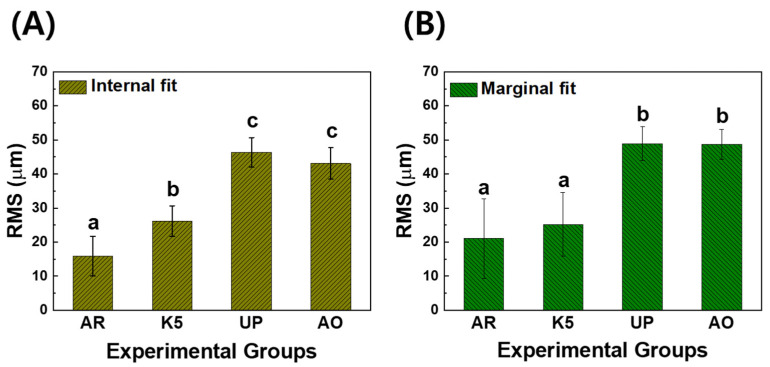
RMS values of (**A**) the internal fit and (**B**) the marginal fit of the zirconia specimens manufactured by subtractive manufacturing and additive manufacturing. In each graph, the values of experimental groups with the same lowercase letter (a, b, or c) were not statistically different, as determined by one-way ANOVA at α = 0.05.

Figure 3 shows the RMS values of the inner and marginal areas of the experimental groups. The RMS values of the internal area showed the superior accuracy of the specimens in the following order: AR (15.89 ± 5.81 μm), K5 (26.17 ± 4.49 μm), AO (40.41 ± 1.25 μm), and UP (46.35 ± 4.33 μm). From the statistical analysis, we noted that the RMS values of the AR group were significantly lower than those of other groups (*p* < 0.05). Moreover, the RMS evaluation of the marginal area demonstrated superior fitting accuracy in the following order: AR (21.06 ± 11.71 μm), K5 (25.20 ± 9.38 μm), AO (48.75 ± 4.39 μm), and UP (48.93 ± 4.90 μm). The RMS values of the AR and K5 groups were significantly lower than those of the AO and UP groups (*p* < 0.05).

**Figure 4:**
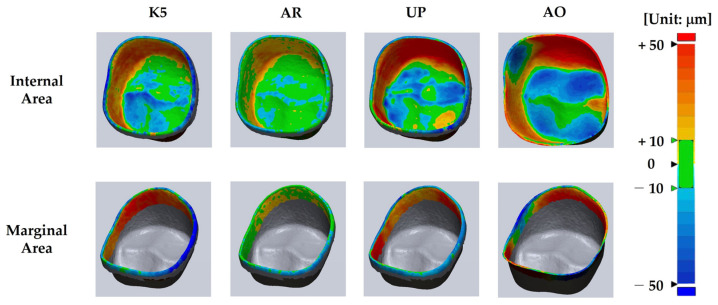
Representative superimposed images of the reference specimen and fabricated specimen (superimposed images of all the experimental specimens are shown in Figure S1).

Figure 4 shows the superimposed images of the zirconia specimens prepared using the three milling machines and one 3D printer. The color of the AR group was more evenly distributed within a range of ~10 mm in the inner and marginal regions than in the other experimental groups. On the contrary, color variations in the internal and marginal areas of the AO and UP groups were higher than those in the K5 and AR groups. In addition, from the overlapped images of the AO and UP groups, a large portion of intaglio (blue color: −50 mm) was observed in the occlusal region, whereas a large portion of embossing (red color: +50 mm) was detected in the occlusal-axial area.

### 3.2. Bond Strength Test of the Veneered Porcelain with Zirconia

**Figure 5:**
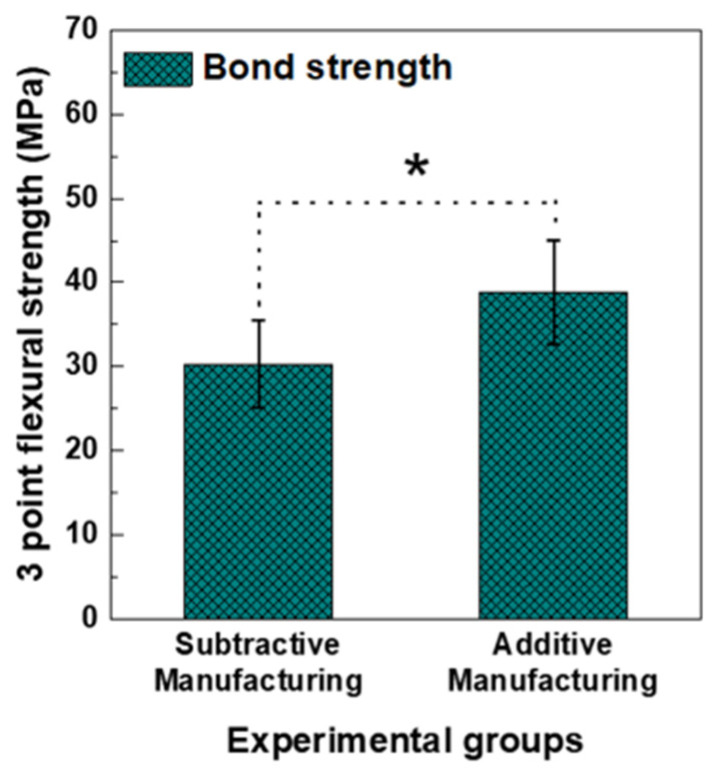
Results of the debonding/crack initiation test between porcelain and zirconia that was block fabricated by subtractive manufacturing and additive manufacturing. The asterisk indicates that there was a significant difference between experimental groups as determined by a *t*-test at α = 0.05.

Figure 5 shows the bond strength results of the veneered porcelain to the zirconia prepared using the AM and SM methods. The bond strength value of the AM-fabricated zirconia group (35.12 ± 4.09 MPa) was significantly higher than that of the SM-fabricated zirconia group (30.26 ± 5.20 MPa) (*p* < 0.05).

**Figure 6:**
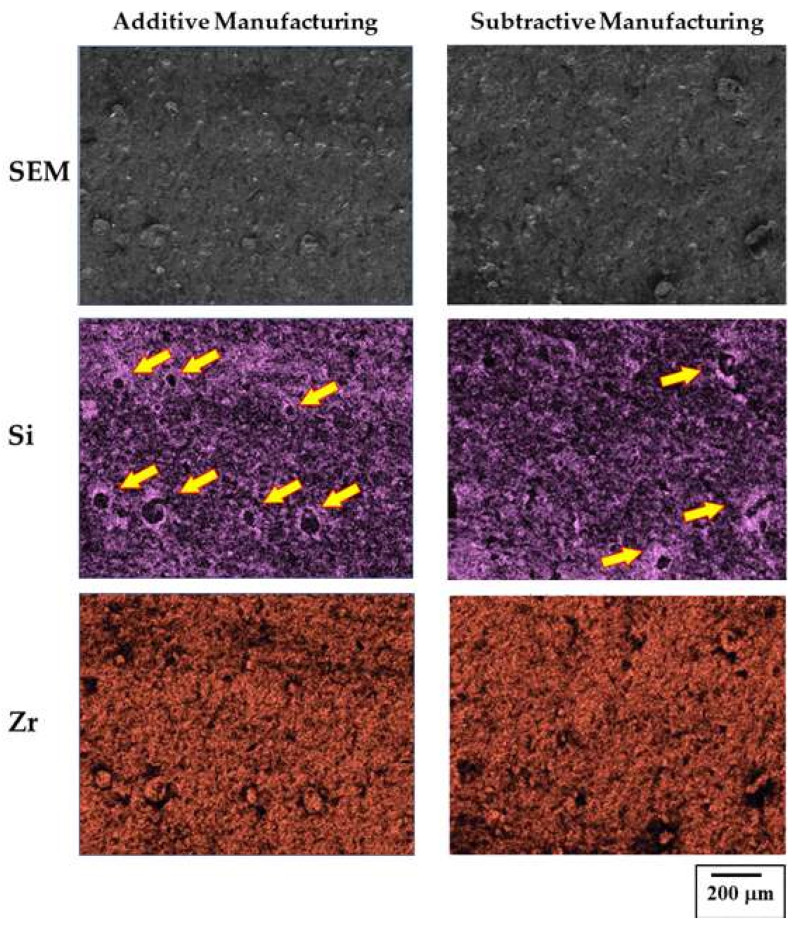
SEM and element (Si and Zr) mapping images of the zirconia specimens prepared via additive manufacturing and subtractive manufacturing.

The SEM results obtained by observing the debonded surface indicated the formation of more dimples on the surface of the AM-fabricated zirconia specimens (Figure 6), which were porcelain components that exhibited a strong bond with the zirconia. We confirmed that the number of dimples in the AM zirconia specimen was higher than that in the SM zirconia specimen.

## 4. Discussion

In this study, we aimed to evaluate the feasibility of using zirconia prepared via AM technology in dental practice. To this end, we assessed the manufacturing accuracy of the dental prostheses completed with the final sintering. Most studies that examined the machining accuracy evaluated the accuracy of the results after machining processes that did not include the sintering process; thus, they were based on purely machined samples. However, the sintering process, which compensates for approximately 20~30% of the thermal shrinkage, is required in general to obtain the final dental product in SM technology. Therefore, this study, which evaluated the manufacturing accuracy using specimens completed with final sintering, fills a gap in the literature.

The results of the RMS values of the zirconia specimens prepared via AM and SM indicated that the AR and UP groups, which were SM groups, showed the best and worst accuracy in the internal (occlusion and occlusion-axial) and marginal areas among all experimental groups, respectively. Moreover, the accuracy of the AO group (AM group) was within the range of accuracy of the three SM groups. During the manufacturing process using the milling machines (SM devices), several factors, such as the directions of the tool paths, type of milling conditions and burrs, and the software that was used for CAM, influenced the deviation of the accuracy of the dental products [28,29,30]. In this study, we used the same zirconia blank and three milling machines to test the accuracy; thus, the compatibility between the zirconia block and the milling machine was also expected to play a critical role when preparing a specimen with high accuracy. Therefore, this variable appeared to cause different deviations in the accuracy of the zirconia specimens manufactured using the three different milling machines.

AM also includes several variables that affect the dimensional accuracy of the final product in the digital CAD/CAM systems during scanning, design, fabrication, and sintering [31]. Among these variables, the laminating directions of 3D printed zirconia are significant factors affecting the manufacturing accuracy and mechanical strength [21]. However, in this study, only one direction (perpendicular to the occlusal plane) of 3D printing was used to fabricate the zirconia specimen. Therefore, further investigation is required to assess the relationship between the layering direction of the 3D printing and the characteristics of the fabricated zirconia specimen. In addition, thermal shrinkage due to the sintering process significantly affects the dimensional accuracy of final dental products [32,33,34]. The slurry-based ceramic 3D printing technology requires an additional heat treatment step to remove the polymer matrix and a ceramic sintering step. Thus, when compared to the SM technique, the AM technique requires more heat treatment and is accompanied by greater thermal shrinkage. Furthermore, the characteristics of the DLP 3D printer used in this study seemed to influence the accuracy of the internal and marginal fits of the fabricated zirconia specimens. A DLP 3D printer has the advantage of being able to quickly manufacture a large area [35]. However, because the laser beam is expanded through a lens to transfer a large-area pattern mask, the straightness of the laser beam is influenced by lens distortion, resulting in a reduction in the accuracy of the final product [36]. Therefore, the step for the additive buildup of the DLP 3D printer is speculated to be one of the shortcomings in achieving precisely manufactured dental products. The overall improvements in the AM process such as the reduction in sintering periods, the simplification of the post-treatment process, and the enhancement of the XY resolution of the 3D printer were considered to improve the manufacturing accuracy of dental prostheses [37,38].

The bond strength test showed that the bond strength in the AM zirconia group was significantly higher than that in the SM zirconia group (*p* < 0.05). In this test, we failed to directly fabricate a zirconia specimen for the three-point flexural strength test using both the AM and SM methods. As the test specimen was significantly thin (0.5 mm), fracture and deformation occurred during the manufacturing process; thus, we prepared large zirconia blocks in advance and then cut them into the final test specimen. This fabrication procedure excluded the effect of the difference in the surface roughness of the AM- and SM-fabricated zirconia specimens because we used the same cutting device and polishing process. The bond strength test results indicated that the strength value of the AM group was higher than that of the SM group.

In addition, we could predict the bond strength results from the SEM observations. The micro-observations of the debonded surfaces showed predominant intragranular fractures on the surface, which were caused by brittle factures in the ceramics. Brittle fractures are generally classified into intragranular fractures and intergranular fractures. The former is a fracture pattern that frequently occurs in ceramics, and the latter is common in metals. In addition, a dimple shape was partially observed at the fracture surface (indicated by the yellow arrows in Figure 6). Through EDS elemental analysis, most elements around the dimple area were the main components of porcelain. The generation of the dimple was caused by a gradual fracture absorbing external stress, to some extent, at the interface between the porcelain and zirconia layers. Thus, we hypothesized that the dimple represented a strong bonding force between the zirconia and porcelain [39]. Between the two specimens, the number of dimples on the surface of the AM zirconia specimen was higher; therefore, a higher number of microdimples was expected to improve the bonding of zirconia to porcelain, which would result in high strength values in the AM group.

From the visual inspection of the specimen, all tested samples were in the typical adhesive failure mode, showing debonding between the porcelain and zirconia after the test. Moreover, all porcelain layers were fractured during the test (data not shown) [40]. As the mechanical strength of zirconia is approximately 10 times higher than that of veneered porcelain [41,42], the adhesive failure of the test specimen seemed to be caused by the different mechanical strengths of zirconia and porcelain. Further investigation is required to analyze the superior bond strength of veneered porcelain to zirconia prepared via AM.

## 5. Conclusions

Within the limitations of this study, the manufacturing accuracy of the zirconia specimen produced by the AM device was within the range of that of the zirconia specimen produced by three different SM devices. In addition, the bond strength value of porcelain to zirconia produced by AM technology was higher than that in the specimen fabricated via SM technology. Furthermore, SEM observation confirmed that a higher number dimples—which implies a strong bond between porcelain and zirconia—were found in the AM zirconia specimen. Therefore, we hypothesize that a dental prosthesis based on AM technology has a considerably high potential for use in dental clinics, and additional research is required for its practical application in dentistry.

## Figures and Tables

**Table 1:** Devices and materials used in this study.

Group		Model	Manufacturer	Materials
SM ^1^	AR	5X-500L	Arum Co., Daejeon, Korea	Luxen Zirconia 1200 Zr, Dentalmax Co., Seoul, Korea
	K5	K5 Impression	vhf camfacture AG, Ammerbuch, Germany	
	UP	P52	UP 3D Co., Shenzhen, China	
AM ^2^	AO	INNI-II	AON, Gunpo, Korea	INNI-Cera, AON, Gunpo, Korea

^1^ SM: subtractive manufacturing; ^2^ AM: additive manufacturing.

## Supplementary Materials

The following supporting information can be downloaded at: https://www.mdpi.com/article/10.3390/ma15155398/s1, Figure S1: Superimposed images of the CAD reference data and the CAD specimen data of all specimens tested in this study.
