# Supplementary material for: A Comparative Study of Additive and Subtractive Manufacturing Techniques for a Zirconia Dental Product: An Analysis of the Manufacturing Accuracy and the Bond Strength of Porcelain to Zirconia
Source: Materials (Basel). 2022 Aug 5;15(15):5398. doi: 10.3390/ma15155398 (PMC9370019; doi:10.3390/ma15155398)
Supplement: Supplementary file 1 [file materials-15-05398-s001.zip › materials-1741858-supplementary.pdf]

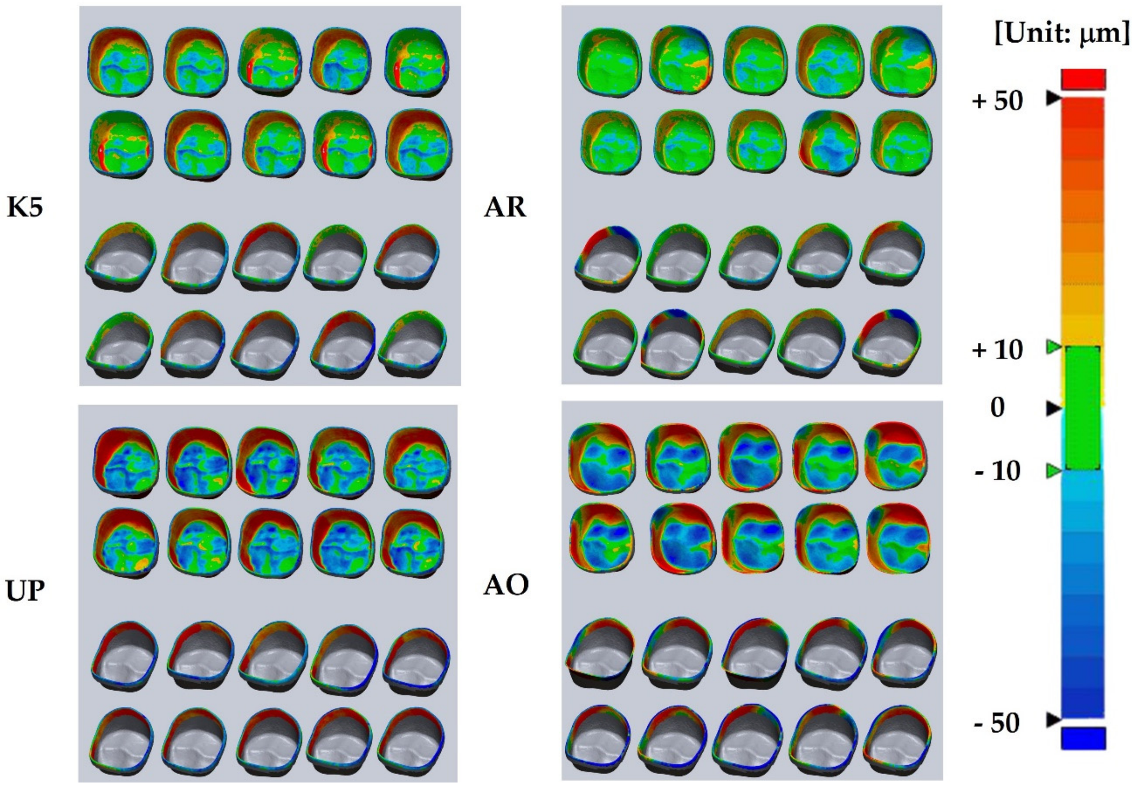

Figure S1. Superimposed images of the CAD reference data and the CAD specimen data of all specimens tested in this study.
